# Intra-operative events during cardiac surgery are risk factors for the development of delirium in the ICU

**DOI:** 10.1186/s13054-016-1445-8

**Published:** 2016-08-21

**Authors:** Alain Rudiger, Hülya Begdeda, Daniela Babic, Bernard Krüger, Burkhardt Seifert, Maria Schubert, Donat R. Spahn, Dominique Bettex

**Affiliations:** 1 Cardio-Surgical Intensive Care Unit, Institute of Anesthesiology, University Hospital Zurich and University of Zurich, Rämistrasse 100, CH-8091 Zurich, Switzerland; 2 Epidemiology, Biostatistics and Prevention Institute (EBPI), Department of Biostatistics, University of Zurich, Hirschengraben 84, CH-8001 Zurich, Switzerland; 3 Centre of Clinical Nursing Science, University Hospital Zurich, Rämistrasse 100, CH-8091 Zurich, Switzerland; 4 Directorate of Nursing/MTT, Inselspital, University Hospital, Freiburgstrasse, 3010 Bern, Switzerland; 5 Institute of Anesthesiology, University Hospital Zurich and University of Zurich, Rämistrasse 100, CH-8091 Zurich, Switzerland

**Keywords:** Cardiac surgery, Cardiopulmonary bypass, Platelets, Transfusion, Risk factors, Delirium, Intensive care unit

## Abstract

**Background:**

Risk factors for delirium following cardiac surgery are incompletely understood. The aim of this study was to investigate whether intra-operative pathophysiological alterations and therapeutic interventions influence the risk of post-operative delirium.

**Methods:**

This retrospective cohort study was performed in a 12-bed cardiosurgical intensive care unit (ICU) of a university hospital and included patients consecutively admitted after cardiac surgery during a 2-month period. The diagnosis of delirium was made clinically using validated scores. Comparisons between patients with and without delirium were performed with non-parametric tests. Logistic regression was applied to identify independent risk factors. Results are given as number (percent) or median (range).

**Results:**

Of the 194 consecutive post-cardiac surgery patients, 50 (26 %) developed delirium during their ICU stay. Univariate analysis revealed that significant differences between patients with and without delirium occurred in the following intra-operative variables: duration of cardiopulmonary bypass (184 [72–299] vs 113 (37–717) minutes, *p* < 0.001), lowest mean arterial pressure (50 [30–70] vs 55 [30–75] mmHg, *p* = 0.004), lowest haemoglobin level (85 [56–133] vs 98 [53–150] g/L, *p* = 0.005), lowest body temperature (34.5 [24.4–37.2] vs 35.1 [23.9–37.2] °C, *p* = 0.035), highest noradrenaline support (0.11 [0.00–0.69] vs 0.07 [0.00–0.42] μg/kg/minute, *p* = 0.001), and frequency of red blood cell transfusions (18 [36 %] vs 26 [18 %], *p* = 0.018) and platelet transfusions (23 [46 %] vs 24 [17 %], *p* < 0.001). Only platelet transfusions remained an independent risk factor in the multivariate analysis (*p* < 0.001).

**Conclusions:**

In patients undergoing cardiac surgery, various intra-operative events, such as transfusion of platelets, were risk factors for the development of a post-operative delirium in the ICU. Further research is needed to unravel the underlying mechanisms.

## Background

Delirium is an acute cognitive dysfunction defined by fluctuating inattention, confusion and pathological changes in consciousness [1]. It is an unspecific manifestation of acute illness occurring frequently in patients after cardiac surgery [2]. Delirium has been attributed to a longer intensive care unit (ICU) and hospital stay [3, 4]. Because efficacious treatment options are lacking, efforts should be made to prevent delirium [5]. Identification of modifiable variables and patients at risk is crucial to the implementation of preventive measures.

Risk factors for delirium were recently summarized for ICU patients in general [6] and for patients undergoing on-pump cardiac surgery in particular [7]. In the review article by Gosselt et al., strong evidence was reported for the following risk factors: age, cerebrovascular disease, psychiatric impairment and cognitive dysfunction, type of surgery and peri-operative red blood cell (RBC) transfusions [7]. Other intra-operative variables, such as duration of cardiopulmonary bypass (CPB) or intra-operative transfusion of platelets, were inconclusive or were not investigated yet [2, 8–10].

The aims of our research were (1) to provide a detailed description of all patients consecutively admitted to the ICU after cardiac surgery during a 2-month period and (2) to explore the association between intra-operative characteristics and the occurrence of post-operative delirium. We hypothesized that intra-operative pathophysiological alterations and therapeutic interventions are significantly associated with the development of post-operative delirium in cardiac surgery ICU patients. A better understanding of these topics potentially may allow better identification of patients at risk as well as the design of interventional studies.

## Methods

### Design

This retrospective cohort study was a sub-study of the larger Health Service Research project Delir Path in which we evaluated different aspects of delirium in hospitalized patients.

### Setting

The study was performed in the cardiosurgical ICU at the University Hospital Zurich, Switzerland. In the year 2013, 1181 patients were treated in this 12-bed ICU, mostly after cardiac or major vascular surgery. The median Simplified Acute Physiology Score (SAPS) II and median ICU mortality of all admitted patients were 31 and 4.3 %, respectively. The ICU is led by certified specialists in intensive care medicine. Neurologists and psychiatrists are available for expert consultation 24 h/day. The principles of patient management in this particular ICU have recently been summarized [11, 12].

In 2012, a hospital-wide delirium project (Delir Path; University Hospital Zurich) was launched to improve the prevention, recognition and treatment of delirium in hospitalized patients. The project has been elaborated by a multi-disciplinary (neurologists, psychiatrists, intensivists, internists, surgeons) and multi-professional (physicians, nurses) team and covers all aspects from diagnostics to pharmacological treatment. Physicians and nurses were trained via lectures, e-learning courses and at the bedside by instructors. Delirium algorithms are available as pocket cards and electronically on the hospital’s intranet. Screening and diagnostic procedures include the Intensive Care Delirium Screening Checklist (ICDSC) [13] performed by the nurses once per shift and the Confusion Assessment Method for the ICU (CAM-ICU) [14, 15] performed by physicians during the morning shift or when the patient shows an acute change or fluctuating course of mental status.

### Participants

**Fig. 1:**
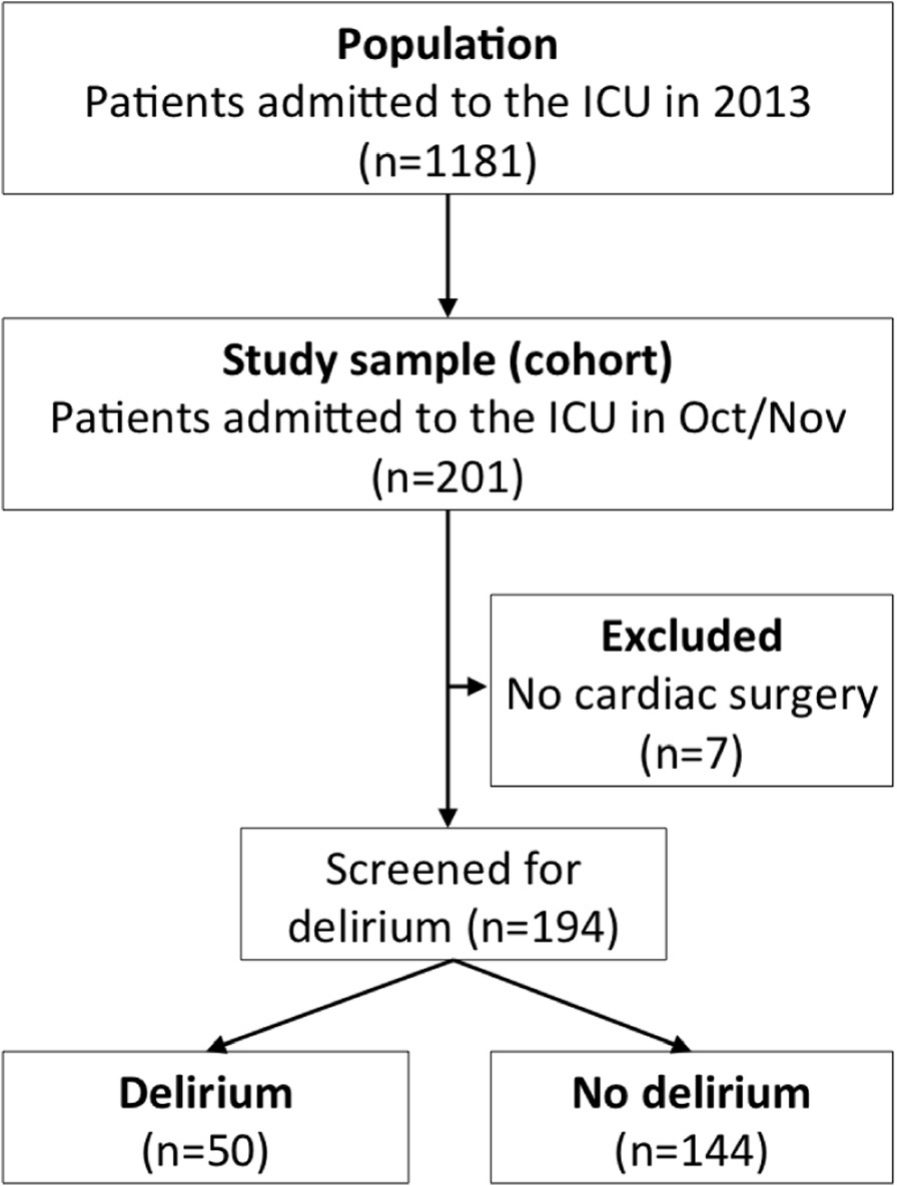
Study flowchart. *ICU* intensive care unit

Data of all patients consecutively admitted to the ICU after cardiac surgery between 1 October and 30 November 2013 were included. Those who did not undergo cardiac surgery were excluded from further analysis (Fig. 1). Patients who developed delirium after ICU discharge, remained in a coma or died in the ICU were neither excluded nor censored from the analysis.

### Delirium (main outcome variable)

Patients with delirium were identified when either the ICDSC was ≥4 and/or the CAM-ICU was positive. The pooled sensitivities and specificities for the detection of delirium in critically ill patients are 80 % and 75 % for the ICDSC and 76 % and 96 % for the CAM-ICU, respectively [16]. In the present study, a positive screening initiated a diagnostic workup leading to the confirmation (or refusal) of the delirium diagnosis. The motor subtype was classified as hypoactive, hyperactive or mixed according to the patient’s behaviour. The delirium diagnosis was documented in the patient’s medical notes and prompted appropriate pharmacological and non-pharmacological treatments. According to the local recommendations, the anti-delirium drug of first choice was pipamperone. This neuroleptic is available as tablets or as syrup and has a weak anti-psychotic effect but a strong sedative action. Haloperidol was added orally or intravenously if hallucinations became predominant. Vegetative symptoms were treated with clonidine or dexmedetomidine intravenously. In selected delirious patients with nocturnal agitation, insomnia or a risk of a non-convulsive epilepsy, intravenous midazolam was continuously infused at doses of 0.05–0.1 mg/kg/h and interrupted daily at 6:00 a.m.

### Predictors

Intra-operatively, information on the lowest mean arterial pressure (MAP), haemoglobin concentration and body temperature, as well as the highest noradrenaline requirement, was collected from the anaesthesia protocol. In addition, the numbers of RBC and platelet transfusions given intra-operatively were documented. Post-operatively, either the highest (leucocyte count, C-reactive protein, creatinine, alanine aminotransferase, lactate) or the lowest (haemoglobin) values upon ICU admission and on day 1, day 2 and day of delirium diagnosis were collected from routine blood samples. Lactate and haemoglobin were measured in the arterial blood by using point-of-care devices (ABL 825 Flex blood gas analyser; Radiometer, Copenhagen, Denmark). All other blood tests were performed in the hospital laboratory.

### Potential confounders

The EuroSCORE II was computed using the calculator available at http://www.euroscore.org/calc.html (EuroSCORE Study Group 2011) [17]. Urgency of cardiac surgery was defined according to the EuroSCORE II definitions as elective (routine admission for operation) and non-elective (urgent, emergency, or salvage surgery) [17]. The SAPS II score was calculated with the worst values during the first 24 h of the ICU stay, with higher values indicating more severe illness and higher predicted mortality [18].

### Statistical methods

Baseline characteristics were determined for the overall population of included patients. Subsequent group comparisons were made between patients who developed delirium during their ICU stay and patients who did not. Values are given as median (range) or number (percent), as appropriate. Groups were compared using the Mann-Whitney *U* test, Pearson’s chi-square test or Fisher’s exact test, as appropriate. The predictive performance of a variable was analysed with the ROC curve. The results were displayed as the AUC and its 95 % CI. The following seven significant intra-operative risk factors were entered into the multivariate analysis: duration of CPB, intra-operative transfusion of RBC (yes or no), intra-operative transfusion of platelets (yes or no), lowest haemoglobin level, lowest MAP, lowest body temperature, highest noradrenaline support (square root-transformed). Forward and backward logistic regression was applied to identify independent risk factors. The OR and its 95 % CI was calculated to quantify how strongly the variable was associated with the presence of delirium. The null hypothesis was rejected with a two-sided *p* value <0.05. All analyses were performed with the use of IBM SPSS Statistics, version 22 software (IBM, Armonk, NY, USA).

## Results

Of 194 patients included in the study, 50 (26 %) developed delirium during their ICU stay. Delirium was diagnosed on ICU day 3 (1–12). The CAM-ICU was positive in 21 of 31 (68 %) patients with delirium, and the ICDSC was ≥4 in 45 of 48 (94 %) patients with delirium. The presentation of delirium was hyperactive, hypoactive and mixed in 17 (36 %), 10 (21 %) and 20 (43 %) of the patients with delirium, respectively. Baseline characteristics for the overall population and the subgroups are given in Table 1. The EuroSCORE II was 4.1 % (0.6–59 %) in patients with delirium and 2.1 % (0.5-35 %) in patients without delirium (*p* = 0.007). Cardiac surgery was elective in 23 (50 %) patients with delirium and in 94 (70 %) patients without delirium (*p* = 0.019). The SAPS II scores in patients with and without delirium were 38 (16–84) and 29 (6–118), respectively (*p* = 0.001).

**Table 1:** Baseline characteristics

Variable	All (*n* = 194)	Delirium (*n* = 50)	No delirium (*n* = 144)	*p* Value
Age, years	66 (23–92)	67 (23–86)	65 (25–92)	0.78
Male sex	147 (76 %)	38 (76 %)	109 (76 %)	1.00
Weight, kg	79 (38–170)	77 (47–135)	79 (38–170)	0.64
Height, cm	172 (147–198)	172 (149–185)	172 (147–198)	0.51
History of alcohol abuse				0.65
Suspected	18 (9.5 %)	3 (6.1 %)	15 (11 %)	
Proven	6 (3.2 %)	3 (6.1 %)	3 (2.1 %)	
History of neurological and/or psychiatric disease	39 (23 %)	14 (29 %)	25 (20 %)	0.23
History of delirium	4 (2.1 %)	3 (6.0 %)	1 (0.7 %)	0.056

Values are given as median (range) or number (percent)

**Fig. 2:**
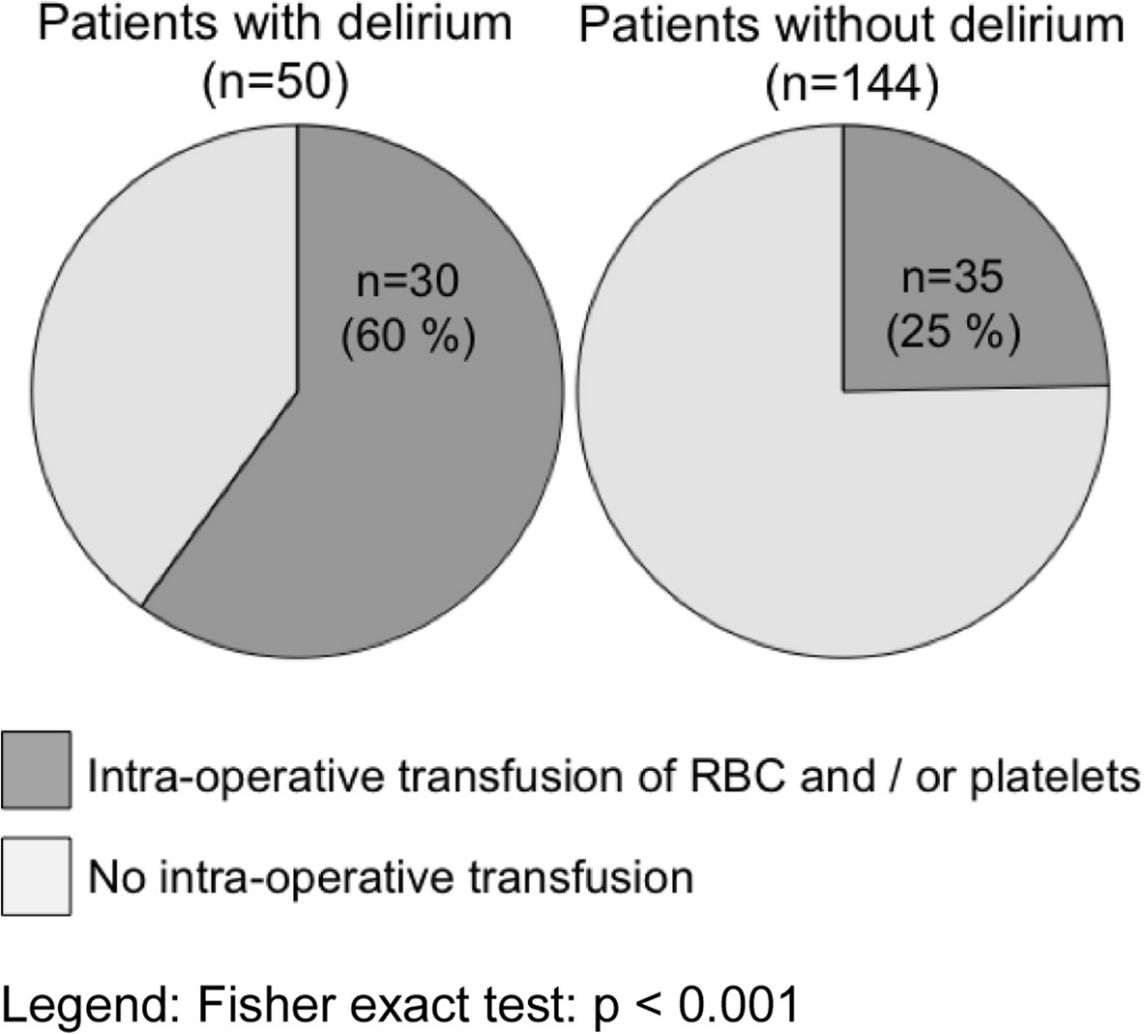
Frequency of intra-operative transfusions of blood products. *p* < 0.001 by Fisher’s exact test. *RBC* red blood cells

Intra-operative characteristics of cardiac surgery are provided in Table 2. As shown in Fig. 2, patients with delirium received RBC and/or platelet transfusions more often (30 [60 %] vs 35 [25 %] patients, respectively; *p* < 0.001). Laboratory values on ICU admission, ICU day 1, ICU day 2 and the day of delirium diagnosis are displayed in Table 3.

**Table 2:** Intra-operative characteristics of cardiac surgery

Variable	All (*n* = 194)	Delirium (*n* = 50)	No delirium (*n* = 144)	*p* Value
Type of cardiac surgery				
CABG surgery	59 (30 %)	11 (22 %)	48 (33 %)	0.50
Valve surgery	69 (36 %)	19 (38 %)	50 (35 %)	
CABG + valve	20 (10 %)	6 (12 %)	14 (9.7 %)	
Other	46 (24 %)	14 (28 %)	32 (22 %)	
Duration of cardiac surgery				
Total, minutes	231 (20–950)	270 (30–565)	217 (20–950)	**0.033**
Non-CPB, minutes (*n* = 194)^a^	130 (15–525)	127 (15–325)	135 (17–525)	0.64
CPB, minutes (*n* = 129)^b^	126 (37–717)	184 (72–299)	113 (37–717)	**<0.001**
ACT, minutes, (*n* = 120)^c^	88 (14–359)	105 (40–221)	80 (14–359)	**0.001**
Intra-operative variables				
Mean arterial pressure, lowest, mmHg	55 (30–75)	50 (30–70)	55 (30–75)	**0.004**
Lowest haemoglobin, lowest, g/L	93 (53–150)	85 (56–133)	98 (53–150)	**0.005**
Lowest body temperature, lowest, °C	35.0 (23.9–37.2)	34.5 (24.4–37.2)	35.1 (23.9–37.2)	**0.035**
Intra-operative vasopressor support				
Noradrenaline, highest, μg/kg/minute	0.08 (0.00–0.69)	0.11 (0.00–0.69)	0.07 (0.00–0.42)	**0.001**
Intra-operative transfusions				
RBC, %	44 (23 %)	18 (36 %)	26 (18 %)	**0.018**
RBC, number of bags (*n* = 44)	3 (1–5)	2 (2–5)	3 (1–5)	0.87
Platelets, %	47 (25 %)	23 (46 %)	24 (17 %)	**<0.001**
Platelets, number of bags (*n* = 47)	1 (1–3)	1 (1–2)	2 (1–3)	0.11
Post-operative LVEF, %	55 (10–76)	55 (20–76)	55 (10–75)	0.59

*Abbreviations: ACT* aortic clamp time, *CABG* coronary artery bypass graft, *CPB* cardiopulmonary bypass, *LVEF* left ventricular ejection fraction, *RBC* red blood cells

Values are given as median (range) or number (percent)

^a^ Duration of non-CPB surgery was calculated as total duration of cardiac surgery minus duration of CPB. In 65 patients without CPB, duration of non-CPB surgery was equal to the total duration of cardiac surgery

^b^ CPB was required in 36 (72 %) patients with delirium and in 93 (65 %) patients without delirium (*p* = 0.60)

^c^ Aortic clamping was performed in 33 (66 %) patients with delirium and in 87 (61 %) patients without delirium (*p* = 0.61)

Bold data indicate significant (p<0.05)

**Table 3:** Laboratory values

Variable	Total (*n* = 194)	Delirium (*n* = 50)	No delirium (*n* = 144)	*p* Value
Haemoglobin, g/L				
ICU admission	97 (45–160)	93 (45–136)	98 (68–160)	**0.047**
ICU day 1	94 (60–142)	85 (63–130)	97 (60–142)	**<0.001**
ICU day 2	87 (60–145)	81 (60–130)	91 (61–145)	**<0.001**
Day of diagnosis	–	81 (65–116)	–	–
Leucocyte count, 10^3^/μl				
ICU admission	11.5 (3.0–54.2)	13.6 (3.4–54.2)	10.9 (3.0–28.0)	**0.035**
ICU day 1	10.3 (4.5–30.3)	10.9 (5.6–28.0)	10.0 (4.5–30.3)	0.21
ICU day 2	10.6 (1.3–31.0)	11.3 (1.3–31.0)	10.2 (2.9–28.0)	0.26
Day of diagnosis	–	9.9 (4.5–23.7)	–	–
C-reactive protein, mg/L				
ICU admission	3.1 (0.3–446)	4.5 (0.3–439)	2.7 (0.3–446)	**0.025**
ICU day 1	58 (0.7–542)	68 (3.9–542)	56 (0.7–478)	0.22
ICU day 2	150 (4.2–497)	150 (4.2–497)	150 (12.0–387)	0.95
Day of diagnosis	–	101 (0.5–497)	–	–
Creatinine, μmol/L				
ICU admission	84 (44–500)	90 (49–500)	83 (44–257)	0.064
ICU day 1	88 (38–382)	96 (41–382)	84 (38–238)	**0.003**
ICU day 2	84 (38–376)	95 (42–376)	80 (38–348)	**0.017**
Day of diagnosis	–	100 (35–445)	–	–
Alanine aminotransferase, U/L				
ICU admission	20 (5–1368)	25 (8–1368)	20 (5–1215)	**0.024**
ICU day 1	23 (8–1774)	29 (11–628)	23 (8–1774)	**0.040**
ICU day 2	25 (8–1572)	30 (8–413)	24 (8–1572)	**0.037**
Day of diagnosis	–	31 (11–926)	–	–
Lactate, mmol/L				
ICU admission	2.6 (0.6–18.0)	3.9 (0.8–16.0)	2.3 (0.6–18.0)	**<0.001**
ICU day 1	2.5 (0.6–19.0)	3.6 (0.9–17.0)	2.3 (0.6–19.0)	**0.008**
ICU day 2	1.6 (0.5–20.0)	1.6 (0.6–8.1)	1.6 (0.5–20.0)	0.31
Day of diagnosis	–	1.6 (1.1–12.8)	–	–

*ICU* intensive care unit

Values are given as median (range). Values are the lowest (haemoglobin) or the highest (leucocyte count, C-reactive protein, creatinine, alanine aminotransferase, lactate) values during 1 day

Bold data indicate significant (p<0.05)

Mechanical ventilation was applied in 180 (93 %) patients, with no difference between the groups (*p* = 1.00). However, total duration of mechanical ventilation was longer in patients with delirium than in patients without delirium (35 [3–663] vs 8 [1–880] h; *p* < 0.001). Noradrenaline was required by 181 (93 %) patients, without a difference between the groups (*p* = 0.74). However, the maximum noradrenaline dose was higher in patients with delirium both intra-operatively (Table 2) and post-operatively (0.17 [0.04–0.67] vs 0.10 [0.01–0.63]; *p* < 0.001). Continuous venovenous haemodialysis was applied in nine (18 %) patients with delirium and in seven (4.8 %) patients without delirium (*p* = 0.013).

ICU length of stay was 5 (1–34) days in patients with delirium and 2 (1–22) days in patients without delirium (*p* < 0.001). ICU mortality rates in patients with delirium and in those without delirium were 4.0 % and 5.6 % (*p* = 1.00), respectively. Of the 144 patients without delirium during their ICU stay, 12 (8.3 %) developed delirium after ICU discharge. Thirty-day mortality was 6.0 % in patients with delirium and 8.3 % in those without delirium (*p* = 1.00). The multivariate analysis with intra-operative variables as potential risk factors for post-operative delirium revealed equal results for the backward and forward logistic regression (Table 4).

**Table 4:** Intra-operative risk factors for post-operative delirium

Variables included in multivariate analysis	Univariate analysis		Multivariate analysis	
	AUCROC (95 % CI)	*p* value^a^	OR (95 % CI)	*p* value^b^
Duration of cardiopulmonary bypass	0.73 (0.64–0.83)	**<0.001**	–	0.29
Lowest intra-operative MAP, mmHg	0.64 (0.54–0.73)	**0.004**	–	0.054
Lowest intra-operative haemoglobin, g/L	0.64 (0.55–0.72)	**0.005**	–	0.29
Lowest intra-operative temperature, °C	0.60 (0.50–0.70)	**0.035**	–	0.84
Highest noradrenaline dose, μg/kg/minute	0.66 (0.57–0.74)	**0.001**	–	0.10
Need for ≥1 RBC transfusion	0.59 (0.49–0.68)	**0.063**	–	0.65
Need for ≥1 platelet transfusion	0.65 (0.55–0.74)	**0.002**	3.9 (1.9–8.0)	**<0.001**

*MAP* mean arterial blood pressure, *RBC* red blood cells

The multivariable analysis (backward logistic regression) revealed that the intra-operative transfusion of one or more bags of platelets was an independent risk factor for the occurrence of delirium after cardiac surgery

^a^ Mann-Whitney *U* test

^b^ *p* > 0.05 from variables not in the equation

Bold data indicate significant (p<0.05)

## Discussion

In this detailed analysis of 194 consecutive patients undergoing cardiac surgery, 26 % of the patients developed delirium in the ICU. While baseline characteristics and type of surgery were similar between groups, patients with and without delirium differed significantly in disease severity scores (SAPS II, EuroSCORE II), intra-operative variables, post-operative laboratory parameters and the use of ICU resources. With regard to intra-operative variables, long CPB duration, low MAP, low haemoglobin level, low body temperature, high noradrenaline requirement, and transfusion of RBCs and platelets were significant intra-operative risk factors. In the multivariate analysis, only platelet transfusions remained independent, suggesting that platelet transfusions are an independent risk factor for the development of delirium in the ICU. In addition, a complex interplay between different events occurring during cardiac surgery might promote post-operative delirium.

In our study, one-fourth of cardiac surgery patients developed delirium during their ICU stay. This frequency is very similar to recently published data [2] but less than previous reports [19–21]. The lower frequency might be explained by the fact that several preventive measures have been implemented in routine clinical care of our patients, such as delirium monitoring, daily interruption of sedation and early mobilization [22, 23]. In addition, the few patients remaining comatose or dying without awakening were not censored, which influenced the frequency of delirium in our study.

Several variables collected intra-operatively were significantly different in patients with delirium and in patients without it. Only platelet transfusion was an independent risk factor, suggesting that a complex interplay of the other intra-operative alterations promotes the development of post-operative delirium. Whether platelet transfusions are a marker for higher severity of illness or whether they play a true pathophysiological role in the development of delirium remains to be investigated in prospective studies.

In the present study, patients who developed delirium had significantly lower haemoglobin levels intra-operatively, as well as from ICU admission to ICU day 2, despite more frequent RBC transfusions. A positive association between low haemoglobin levels and delirium was found in some studies [24–26], but not in others [27, 28]. Pre-clinical research in rats showed that blood transfusions in general and free haemoglobin in particular increase interleukin 6 levels and cause neuroinflammation with subsequent cognitive dysfunction [29]. Whether measures aimed at decreasing the need for RBC transfusions, such as correction of pre-operative anaemia and particularly meticulous surgical technique and/or additional preventive measures in patients receiving RBC transfusions, are beneficial in patients undergoing long CPB needs to be investigated in future studies. In addition, more research is needed to investigate the role of free haemoglobin as a potential mechanism for delirium after cardiac surgery.

In patients with delirium, post-operative leucocyte counts and C-reactive protein levels were slightly but significantly higher on ICU admission, which might indicate a role of systemic inflammation in the development of delirium. This hypothesis is supported by previous work demonstrating elevated levels of cytokines such as interleukin 6 in patients with delirium [30, 31].

Higher creatinine values on ICU days 1 and 2 and more frequent use of continuous venovenous haemodialysis in patients with delirium indicate an association between post-operative renal dysfunction and delirium, as previously described by others [9]. The recent systematic review by Gosselt et al. described moderate evidence for an association between post-operative renal insufficiency and the development of delirium [7].

### Limitations of the study

The present study is limited by the number of included patients. While the small number of patients enabled a more detailed description of the study participants, it affected the regression analysis because approximately ten patients with delirium are required for every variable included in the multivariate statistics. Our multivariate analysis included only seven intra-operative variables. A bigger database is needed to include additional variables in the regression analysis, particularly to investigate the effects of confounders.

The study is in nature observational but represents accompanying research after the implementation of a multi-professional and multi-disciplinary delirium protocol describing delirium monitoring, prevention and treatment. As we included consecutive patients, few patients died before awakening and thus were unable to develop a delirium. This potentially reduces the group differences found in this study. Others were discharged from the ICU and developed delirium later on the ward. The later patients were not classified as delirious in this report. Hence, our conclusions are valid only for the patients who developed delirium during their ICU stay. Some patients with delirium might have been missed in our study, as the sensitivity of diagnostic screening tools is lower in routine daily practice than in a research setting, particularly for hypoactive delirium [32].

In our study, patients with delirium had a longer ICU stay, which supports previous reports [3, 4]. In contrast to earlier studies [33], ICU mortality in our study was not different between patients with and without delirium. Further research is needed to investigate the use of ICU resources that were necessary to achieve this low mortality.

## Conclusions

Delirium occurred in 26 % of consecutive ICU patients undergoing cardiac surgery. Patients with and without delirium differed significantly in disease severity scores, intra-operative variables, post-operative laboratory parameters and the use of ICU resources. With regard to intra-operative variables, CPB duration, low MAP, low haemoglobin levels, low body temperature, high noradrenaline requirements, and transfusions of RBC and platelets were significant intra-operative risk factors. In the multivariate analysis, only platelet transfusions remained independent. This suggests that the interplay of different events during cardiac surgery might promote post-operative delirium. More research is needed to unravel the underlying mechanisms.

## Key messages

- After cardiac surgery, 26 % of patients developed delirium during their ICU stay.
- Patients with and without delirium differed significantly in intra-operative variables, post-operative laboratory parameters and the use of ICU resources.
- Intra-operative events such as transfusions of blood products are likely to promote post-operative delirium in the ICU.

## Abbreviations

ACT, aortic clamp time; CABG, coronary artery bypass graft; CAM-ICU, Confusion Assessment Method for the ICU; CPB, cardiopulmonary bypass; ICDSC, Intensive Care Delirium Screening Checklist; ICU, intensive care unit; LVEF, left ventricular ejection fraction; MAP, mean arterial pressure; RBC, red blood cell; SAPS, Simplified Acute Physiology Score

## References

[1] Reade MC, Finfer S (2014). Sedation and delirium in the intensive care unit. N Engl J Med..

[2] McPherson JA, Wagner CE, Boehm LM, Hall JD, Johnson DC, Miller LR (2013). Delirium in the cardiovascular ICU: exploring modifiable risk factors. Crit Care Med..

[3] Lat I, McMillian W, Taylor S, Janzen JM, Papadopoulos S, Korth L (2009). The impact of delirium on clinical outcomes in mechanically ventilated surgical and trauma patients. Crit Care Med..

[4] Ely EW, Gautam S, Margolin R, Francis J, May L, Speroff T (2001). The impact of delirium in the intensive care unit on hospital length of stay. Intensive Care Med..

[5] Barr J, Fraser GL, Puntillo K, Ely EW, Gelinas C, Dasta JF (2013). Clinical practice guidelines for the management of pain, agitation, and delirium in adult patients in the intensive care unit. Crit Care Med..

[6] Zaal IJ, Devlin JW, Peelen LM, Slooter AJ (2015). A systematic review of risk factors for delirium in the ICU. Crit Care Med..

[7] Gosselt AN, Slooter AJ, Boere PR, Zaal IJ (2015). Risk factors for delirium after on-pump cardiac surgery: a systematic review. Crit Care..

[8] Gottesman RF, Grega MA, Bailey MM, Pham LD, Zeger SL, Baumgartner WA (2010). Delirium after coronary artery bypass graft surgery and late mortality. Ann Neurol..

[9] Mariscalco G, Cottini M, Zanobini M, Salis S, Dominici C, Banach M (2012). Preoperative statin therapy is not associated with a decrease in the incidence of delirium after cardiac operations. Ann Thorac Surg..

[10] Krzych LJ, Wybraniec MT, Krupka-Matuszczyk I, Skrzypek M, Bolkowska A, Wilczynski M (2013). Complex assessment of the incidence and risk factors of delirium in a large cohort of cardiac surgery patients: a single-center 6-year experience. Biomed Res Int..

[11] Hauffe T, Krüger B, Bettex D, Rudiger A (2015). Shock management for cardio-surgical ICU patients – the golden hours. Cardiac Failure Rev..

[12] Hauffe T, Krüger B, Bettex D, Rudiger A (2016). Shock management for cardio-surgical intensive care unit patients: the silver days. Cardiac Failure Rev..

[13] Bergeron N, Dubois MJ, Dumont M, Dial S, Skrobik Y (2001). Intensive Care Delirium Screening Checklist: evaluation of a new screening tool. Intensive Care Med..

[14] Ely EW, Inouye SK, Bernard GR, Gordon S, Francis J, May L (2001). Delirium in mechanically ventilated patients: validity and reliability of the Confusion Assessment Method for the Intensive Care Unit (CAM-ICU). JAMA..

[15] Ely EW, Margolin R, Francis J, May L, Truman B, Dittus R (2001). Evaluation of delirium in critically ill patients: validation of the Confusion Assessment Method for the Intensive Care Unit (CAM-ICU). Crit Care Med..

[16] Neto AS, Nassar AP, Cardoso SO, Manetta JA, Pereira VG, Esposito DC (2012). Delirium screening in critically ill patients: a systematic review and meta-analysis. Crit Care Med..

[17] Nashef SA, Roques F, Sharples LD, Nilsson J, Smith C, Goldstone AR (2012). EuroSCORE II. Eur J Cardiothorac Surg..

[18] Le Gall JR, Lemeshow S, Saulnier F (1993). A new Simplified Acute Physiology Score (SAPS II) based on a European/North American multicenter study. JAMA..

[19] Rudolph JL, Jones RN, Levkoff SE, Rockett C, Inouye SK, Sellke FW (2009). Derivation and validation of a preoperative prediction rule for delirium after cardiac surgery. Circulation..

[20] Blachy PH, Starr A (1964). Post-cardiotomy delirium. Am J Psychiatry..

[21] Chang YL, Tsai YF, Lin PJ, Chen MC, Liu CY (2008). Prevalence and risk factors for postoperative delirium in a cardiovascular intensive care unit. Am J Crit Care..

[22] Morandi A, Brummel NE, Ely EW (2011). Sedation, delirium and mechanical ventilation: the ‘ABCDE’ approach. Curr Opin Crit Care..

[23] Balas MC, Vasilevskis EE, Olsen KM, Schmid KK, Shostrom V, Cohen MZ (2014). Effectiveness and safety of the awakening and breathing coordination, delirium monitoring/management, and early exercise/mobility bundle. Crit Care Med..

[24] Kazmierski J, Kowman M, Banach M, Fendler W, Okonski P, Banys A (2010). Incidence and predictors of delirium after cardiac surgery: results from the IPDACS Study. J Psychosom Res..

[25] Tully PJ, Baker RA, Winefield HR, Turnbull DA (2010). Depression, anxiety disorders and type D personality as risk factors for delirium after cardiac surgery. Aust N Z J Psychiatry..

[26] Bilotta F, Lauretta MP, Borozdina A, Mizikov VM, Rosa G (2013). Postoperative delirium: risk factors, diagnosis and perioperative care. Minerva Anestesiol..

[27] Bakker RC, Osse RJ, Tulen JH, Kappetein AP, Bogers AJ (2012). Preoperative and operative predictors of delirium after cardiac surgery in elderly patients. Eur J Cardiothorac Surg..

[28] Schoen J, Meyerrose J, Paarmann H, Heringlake M, Hueppe M, Berger KU (2011). Preoperative regional cerebral oxygen saturation is a predictor of postoperative delirium in on-pump cardiac surgery patients: a prospective observational trial. Crit Care..

[29] Tan H, Bi J, Wang Y, Zhang J, Zuo Z (2015). Transfusion of old RBCs induces neuroinflammation and cognitive impairment. Crit Care Med..

[30] van den Boogaard M, Kox M, Quinn KL, van Achterberg T, van der Hoeven JG, Schoonhoven L (2011). Biomarkers associated with delirium in critically ill patients and their relation with long-term subjective cognitive dysfunction: indications for different pathways governing delirium in inflamed and noninflamed patients. Crit Care..

[31] Skrobik Y, Leger C, Cossette M, Michaud V, Turgeon J (2013). Factors predisposing to coma and delirium: fentanyl and midazolam exposure; *CYP3A5*, *ABCB1*, and *ABCG2* genetic polymorphisms; and inflammatory factors. Crit Care Med..

[32] van Eijk MM, van den Boogaard M, van Marum RJ, Benner P, Eikelenboom P, Honing ML (2011). Routine use of the Confusion Assessment Method for the intensive care unit: a multicenter study. Am J Respir Crit Care Med..

[33] Ouimet S, Kavanagh BP, Gottfried SB, Skrobik Y (2007). Incidence, risk factors and consequences of ICU delirium. Intensive Care Med..

